# Supplementary material for: Mechanism of selective recognition of Lys48-linked polyubiquitin by macrocyclic peptide inhibitors of proteasomal degradation
Source: Nat Commun. 2023 Nov 8;14:7212. doi: 10.1038/s41467-023-43025-4 (PMC10632358; doi:10.1038/s41467-023-43025-4)
Supplement: Supplementary file 1 — Supplementary Information [file 41467_2023_43025_MOESM1_ESM.pdf]

Supplementary Information

Mechanism of selective recognition of Lys48-linked polyubiquitin by macrocyclic peptide inhibitors of proteasomal degradation

Betsegaw Lemma, Di Zhang, Ganga B. Vamisetti, Bryan G. Wentz, Hiroaki Suga, Ashraf Brik, Jacek Lubkowski, David Fushman

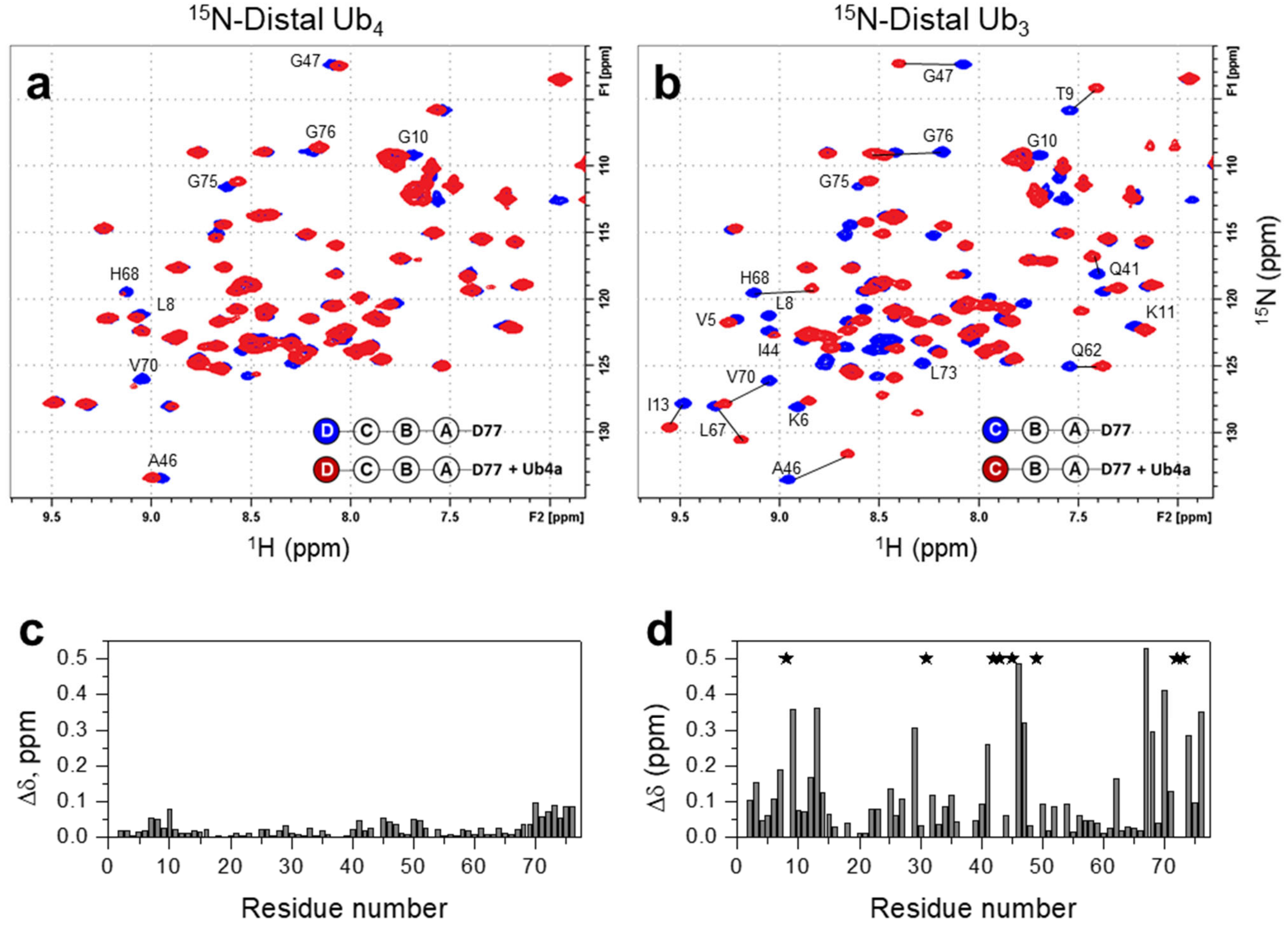

Supplementary Figure 1. The effect of the Ub4a binding to K48-linked tetra-Ub or tri-Ub on the NMR spectra of the ^{15}N-labeled distal Ub unit in these chains. Top: Overlays of ^{1}H-^{15}N SOFAST-HMQC spectra of ^{15}N-labeled distal Ub in (a) tetra-Ub and (b) tri-Ub in the absence (blue) and presence (red) of the cyclic peptide Ub4a. Select residues are indicated. Bottom: Residue-specific chemical shift perturbations (grey bars) in the distal Ub of (c) tetra-Ub and (d) tri-Ub caused by Ub4a binding. Stars (*) mark residues that exhibited strong signal attenuations upon Ub4a binding.

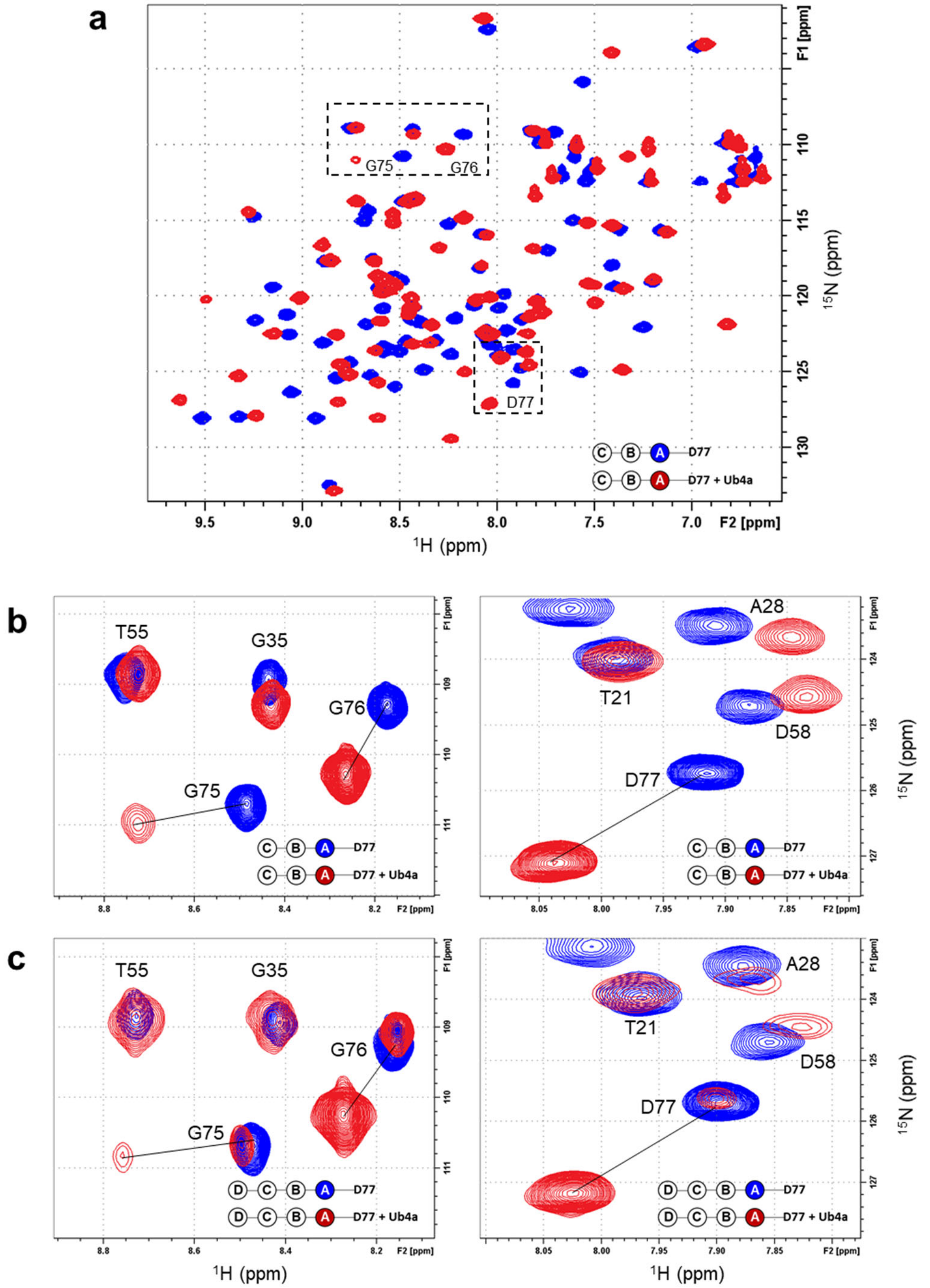

Supplementary Figure 2. Ub4a binding to K48-linked tri- and tetra-Ub causes NMR signal shifts in the C-terminal tail residues of the proximal Ub.

(**a**) Overlay of ^{1}H-^{15}N SOFAST-HMQC spectra of ^{15}N-labeled proximal Ub in K48-linked tri-Ub in the free (blue) and Ub4a-bound (red) states. Dashed rectangles mark regions of the spectrum containing signals of the C-terminal residues G75, G76, and D77.

(**b**, **c**) Zoom in on the rectangles from panel **a**, for (**b**) tri-Ub and (**c**) tetra-Ub. Signals of the tail residues as well as other select residues are indicated. Note that in the spectra shown in panel **c** the Ub4a concentration did not reach saturation, resulting in the signals of both peptide-bound and unbound tetra-Ub species present, as expected for slow exchange on the NMR time scale.

a Amino acid sequences of the proximal-Ub constructs used in this work

WT Ub (residues 1-76):
MQIFVKTLTG KTITLEVEPS DTIENVKAKI QDKEGIPPDQ QRLIFAGKQL EDGRTLSDYN IQKESTLHLV LRLRGG

Ub_{R74} (ΔGG) (residues 1-74):
MQIFVKTLTG KTITLEVEPS DTIENVKAKI QDKEGIPPDQ QRLIFAGKQL EDGRTLSDYN IQKESTLHLV LRLR

Ub_{R72} (ΔLRGG) (residues 1-72):
MQIFVKTLTG KTITLEVEPS DTIENVKAKI QDKEGIPPDQ QRLIFAGKQL EDGRTLSDYN IQKESTLHLV LR

Ub_{D77} (residues 1-76 plus D77)
MQIFVKTLTG KTITLEVEPS DTIENVKAKI QDKEGIPPDQ QRLIFAGKQL EDGRTLSDYN IQKESTLHLV LRLRGGD

b

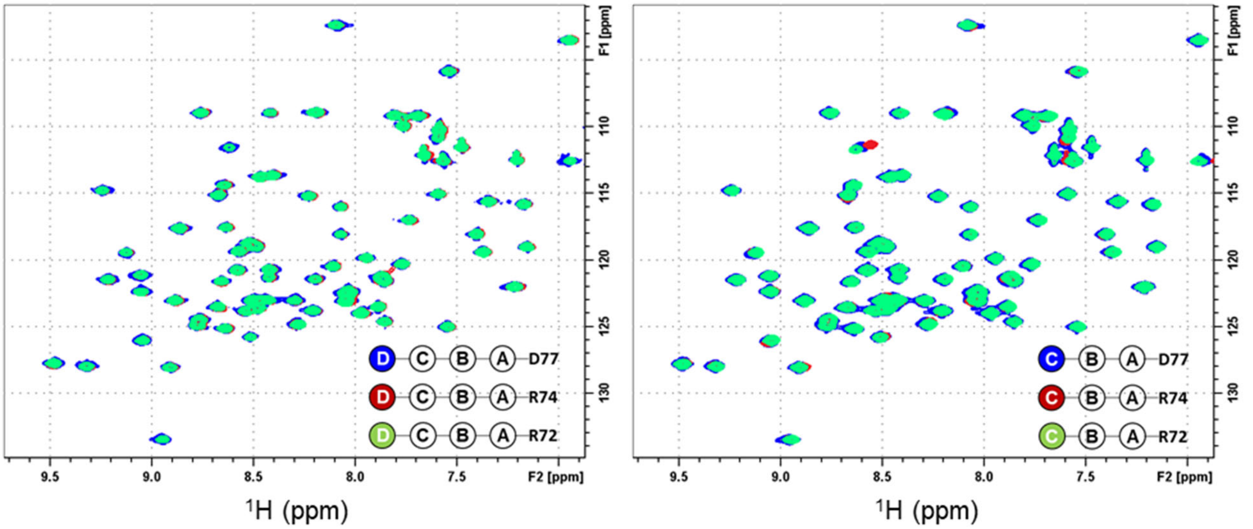

c

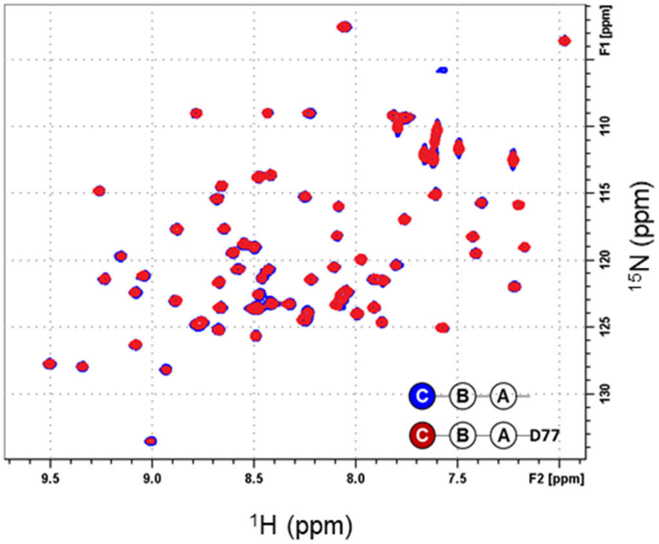

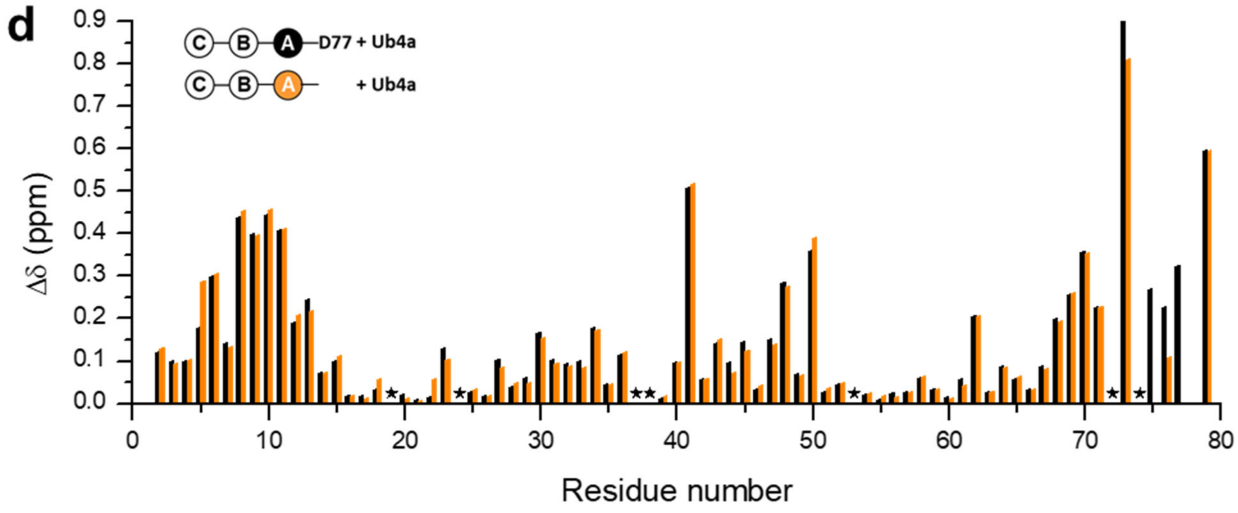

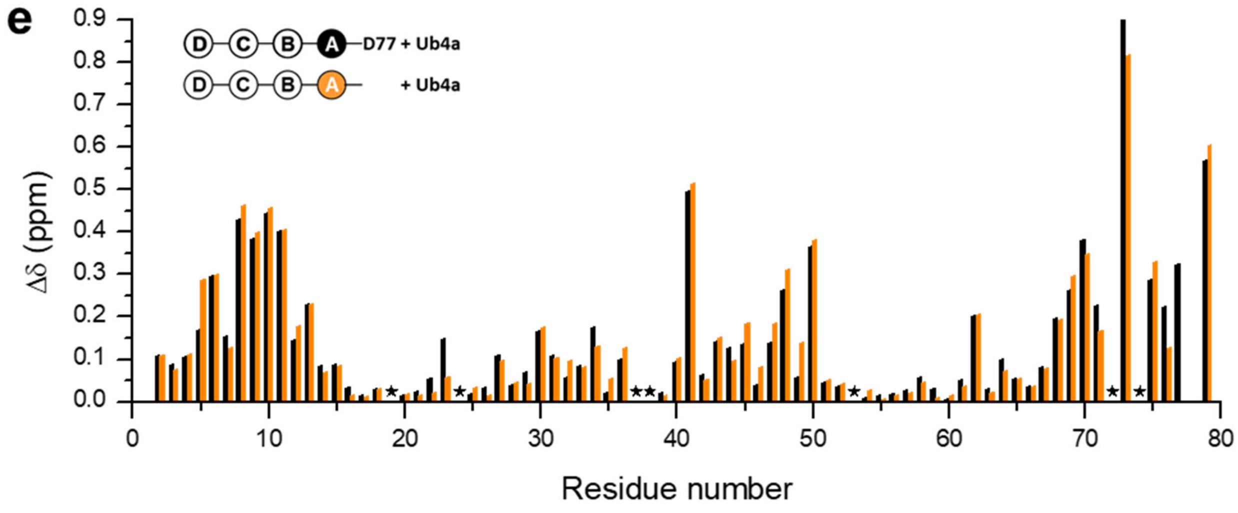

Supplementary Figure 3

(**a**) Amino acid sequences of the proximal-Ub constructs used in this work. The C-terminal tail amino acids L73-G76 are colored blue.

(**b**) C-terminal deletions in the proximal Ub have no or minimal effect on the NMR spectra of the ^{15}N-labeled distal Ub in K48-linked tetra-Ub (left) and tri-Ub (right)

(**c**) C-terminal extension of the proximal Ub by aspartate (D77) has no or minimal effect on the NMR spectra of the ^{15}N-labeled distal Ub in K48-linked tri-Ub

(**d, e**) C-terminal extension of the proximal Ub by aspartate (D77) has no or minimal effect on the chemical shift perturbations in ^{15}N-labeled proximal Ub caused by Ub4a binding to K48-linked tri-Ub (**d**) and tetra-Ub (**e**) as evidenced by comparison of the Ub4a-induced residue-specific CSPs detected in the proximal Ub unit (Ub_A) with D77 (black bars) and without D77 (orange bars). NMR signals of the residues/amides marked with stars could not be observed (prolines) or reliably assigned upon Ub4a binding. The CSPs of the K48 side-chain NH group involved in the isopeptide bond are shown at residue position 79.

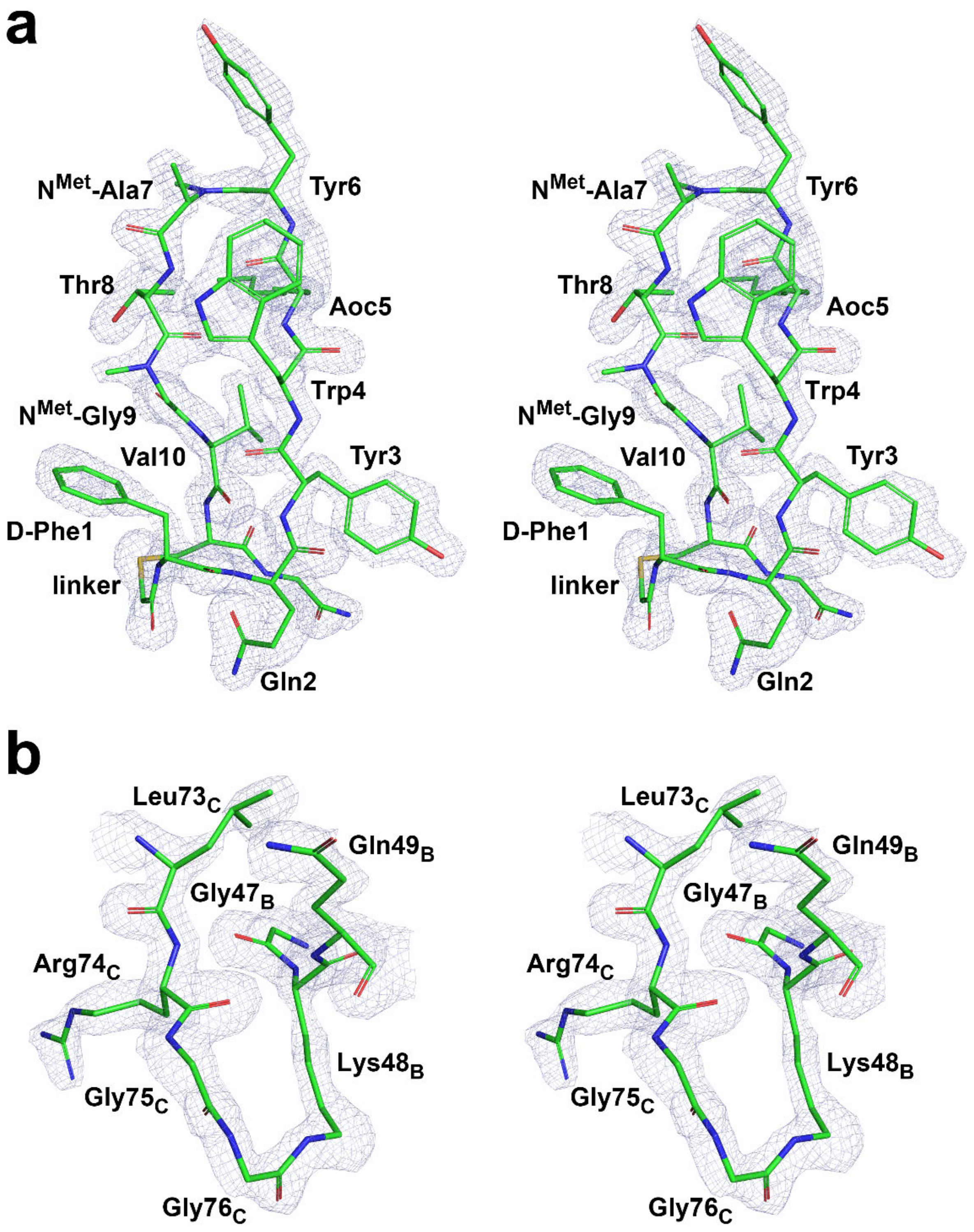
a
N^{Met}-Ala7
Tyr6
Thr8
Aoc5
Trp4
N^{Met}-Gly9
Val10
Tyr3
D-Phe1
linker
Gln2
b
Leu73$_C$
Gln49$_B$
Gly47$_B$
Arg74$_C$
Lys48$_B$
Gly75$_C$
Gly76$_C$

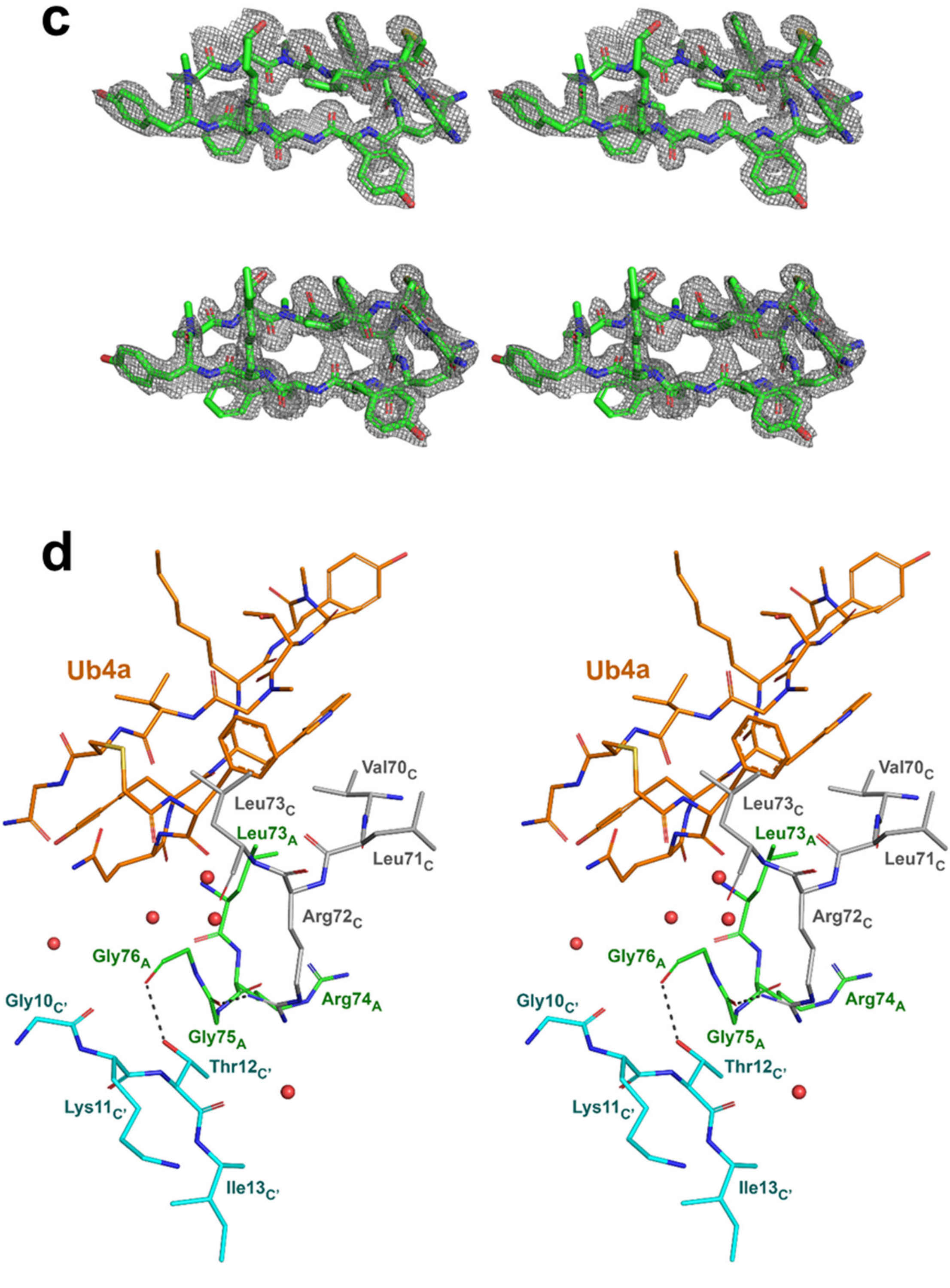

Supplementary Figure 4. Stereoviews of elements of Ub_3:Ub4a crystal structure.

(**a**) Stereoview of the structure of Ub4a in complex with K48-linked tri-Ub. Shown as blue mesh is the $2mF_o$-DF_c electron density map contoured at 1.5 σ level.

(**b**) Stereoview of the fragment of tri-Ub structure in complex with Ub4a, showing the isopeptide linkage between the C-terminus of the distal Ub (Ub_C) and K48 of the middle (*endo*) Ub (Ub_B). Shown as blue mesh is the $2mF_o$-DF_c electron density map contoured at 1.5 σ level.

(**c**) Electron density omit maps shown in stereo projection. (Top) Structure of the cyclic peptide Ub4a with the corresponding mF_o-DF_c omit map for the first complex in the asymmetric unit. (Bottom) Structure of the cyclic peptide Ub4a with the corresponding mF_o-DF_c omit map for the second complex in the asymmetric unit. The omit maps are shown as gray mesh contoured at 3 σ level.

(**d**) Stereoview of the environment of the C-terminus of Ub_A in the crystal. Units A and C from common tri-Ub chain are colored green and gray, respectively. Unit C', symmetry related to Ub_C, is colored blue. Ub4a molecule, colored orange, and several water molecules, represented by red spheres, are also shown. All depicted ubiquitin residues are labeled, the subscript indicates the corresponding ubiquitin unit. Interactions between the C-terminal $Gly76_A$ of Ub_A and $Thr12_{C'}$ of $Ub_{C'}$ as well as between $Gly75_A$ and $Arg72_C$ of Ub_C are indicated with dashed lines.

a

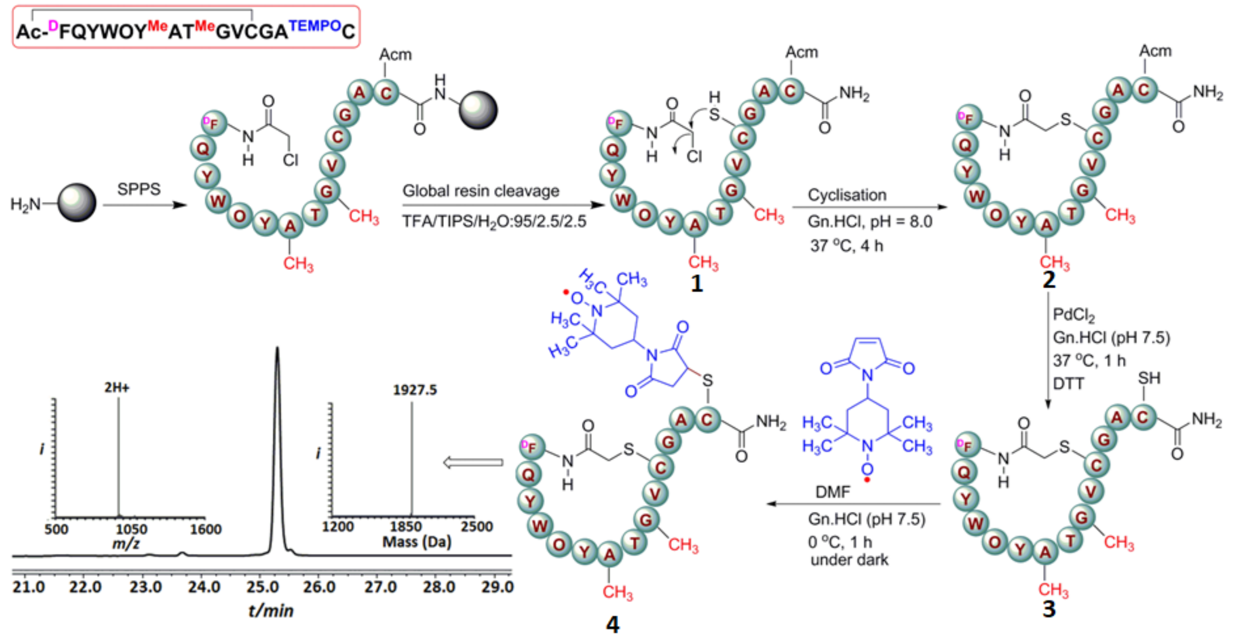

b

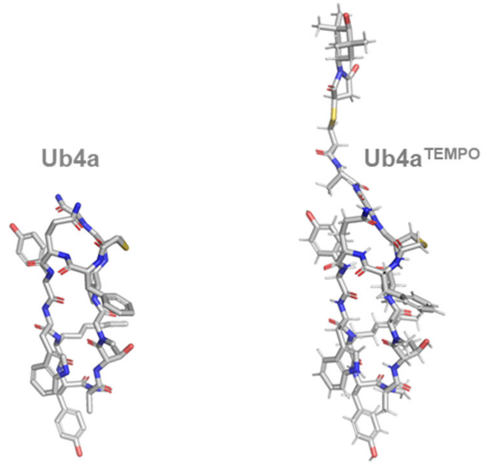

Supplementary Figure 5. Synthesis of Ub4a^{TEMPO} peptide

(**a**) Schematic representation of the synthesis of Ub4a with attached TEMPO free radical (Ub4a^{TEMPO}). Also shown are the results of HPLC-MS analysis of Ub4a^{TEMPO}; the observed mass is 1927.5 ± 0.1 Da, the expected mass is 1927.9 Da (average isotopes). Here "O" represents the Fmoc-L-2-aminooctanoic acid.

(**b**) Crystal structure of Ub4a (from the Ub_3:Ub4a complex) (left) and a modeled structure of Ub4a^{TEMPO} (right).

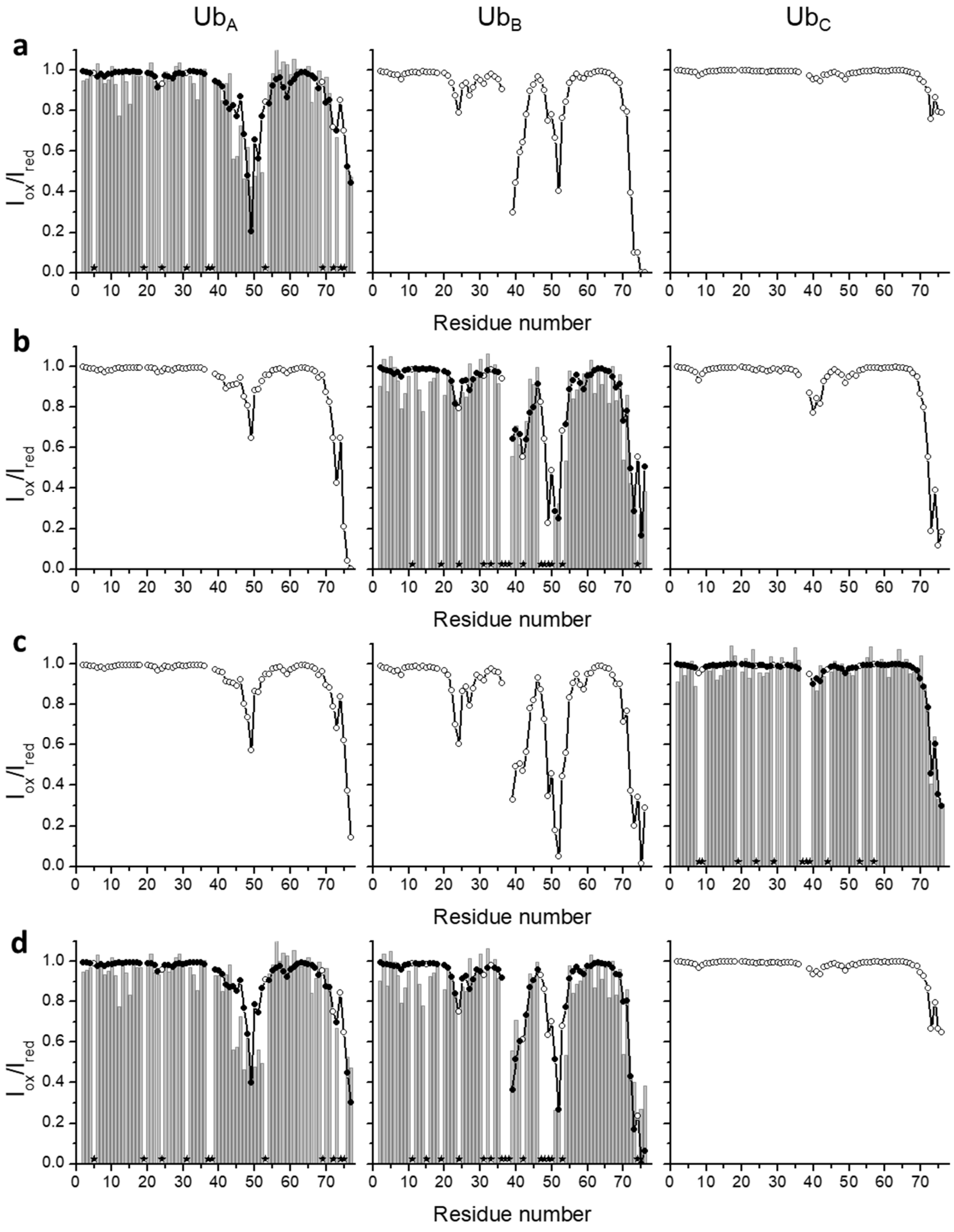

Supplementary Figure 6. NMR/PRE studies of the complex of K48-linked tri-Ub with Ub4a^{TEMPO}

Illustration of PRE data fits for the proximal Ub only (**a**), middle/*endo* Ub only (**b**), distal Ub only (**c**), and proximal+*endo* Ubs together using our crystal structure of the Ub_3:Ub4a complex. The bars depict residue-specific signal attenuations, the solid circles+lines show the results of their fit to a single position of the unpaired electron. The open circles+lines (without vertical bars) represent predicted PRE data for Ub units not used in the fit or for those residues (marked with stars) in Ub units analyzed where the data were not available. The predictions were made using the location of the unpaired electron determined from the corresponding fit and the crystal structure of the Ub_3:Ub4a complex.

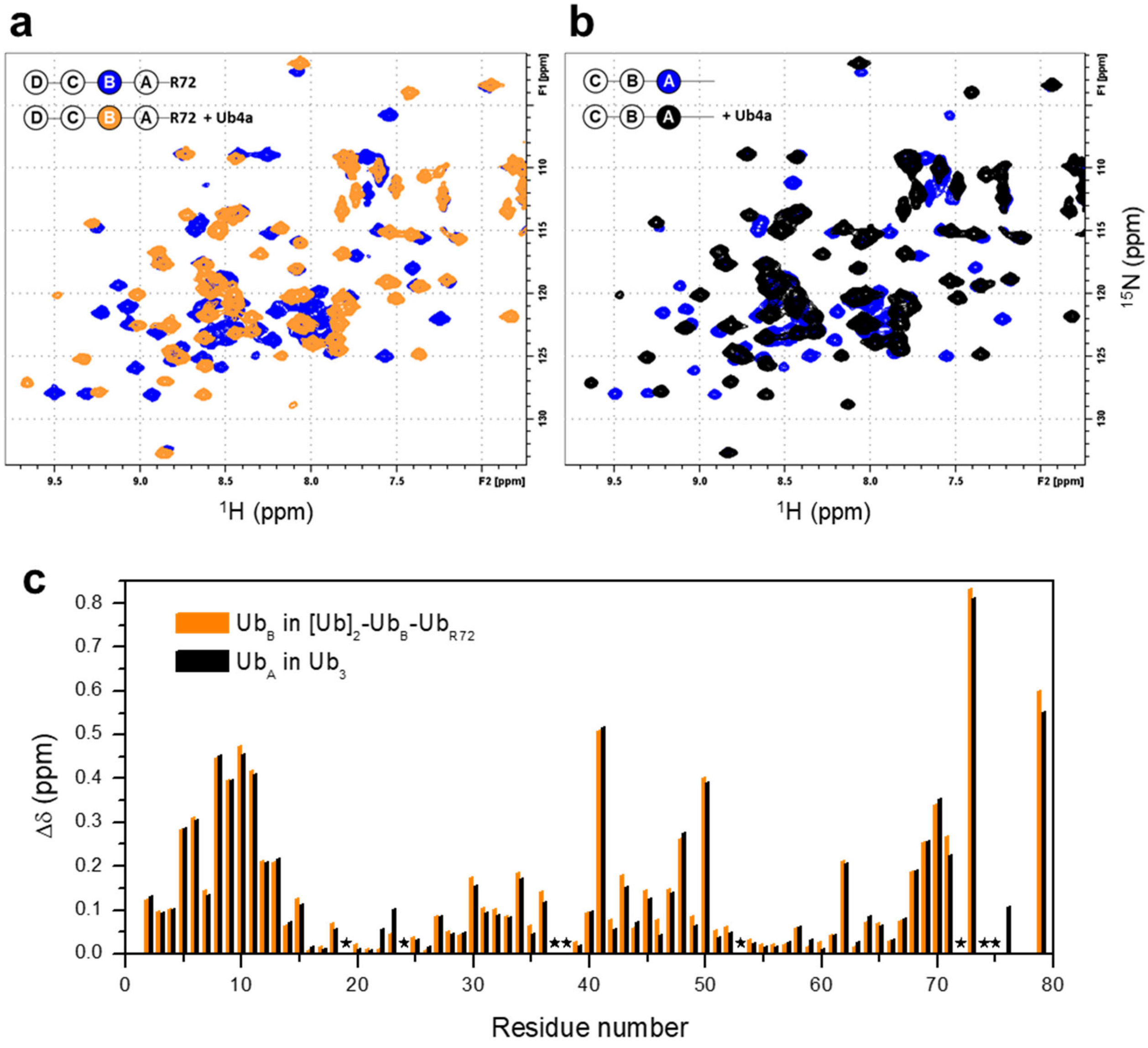

Supplementary Figure 7. NMR spectra indicate similar interactions of Ub4a with the *endo/*next-to-proximal (Ub_B) unit in $[Ub]_2$-$^{15N}Ub_B$-Ub_{R72} and with the proximal Ub (Ub_A) in tri-Ub ($[Ub]_2$-$^{15N}Ub_A$) having natural (un-truncated) C terminus.

(**a**) Overlay of ^{1}H-^{15}N SOFAST-HMQC spectra of $[Ub]_2$-$^{15N}Ub_B$-Ub_{R72} free (blue) and in complex (orange) with Ub4a. (**b**) Overlay of ^{1}H-^{15}N SOFAST-HMQC spectra of $[Ub]_2$-$^{15N}Ub_A$ free (blue) and in complex (black) with Ub4a. (**c**) Comparison of residue-specific chemical shift perturbations (CSPs) caused by Ub4a binding observed in the spectra in panels **a** (orange) and **b** (black). Stars mark residues that could not be observed (prolines) or reliably assigned due to strong signal attenuation upon Ub4a binding. The CSPs of the K48 side-chain NH group involved in the isopeptide bond are shown at residue position 79.

a

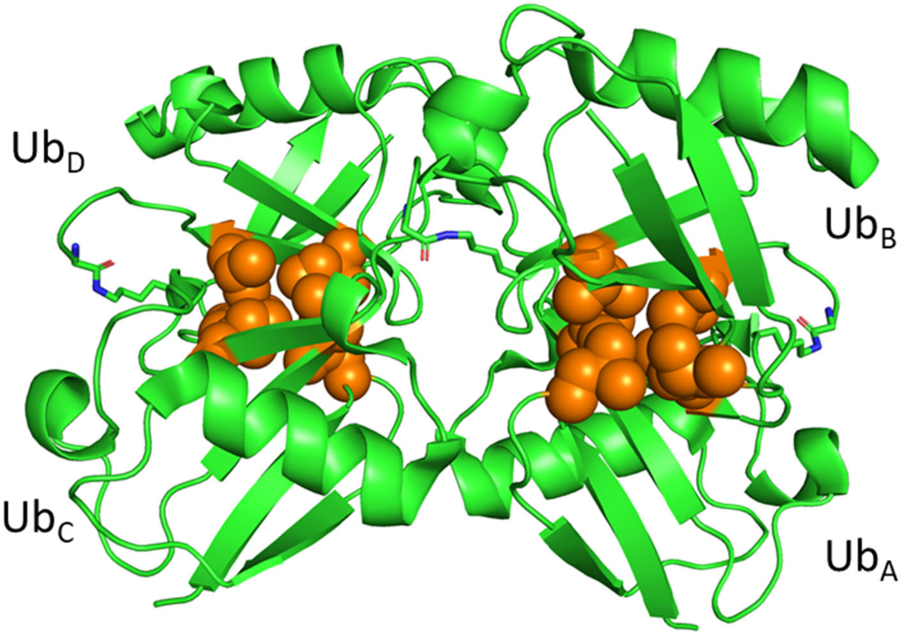

Ub$_D$
Ub$_B$
Ub$_C$
Ub$_A$

b

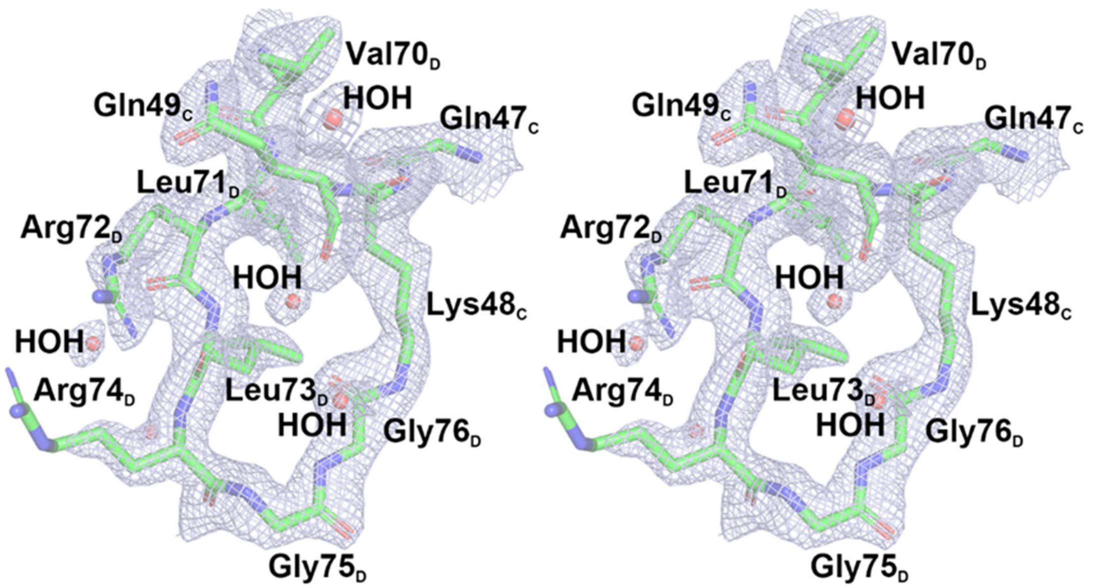

Val70$_D$
HOH
Gln49$_C$
Gln47$_C$
Leu71$_D$
Arg72$_D$
HOH
Lys48$_C$
HOH
Arg74$_D$
Leu73$_D$
HOH
Gly76$_D$
Gly75$_D$

c

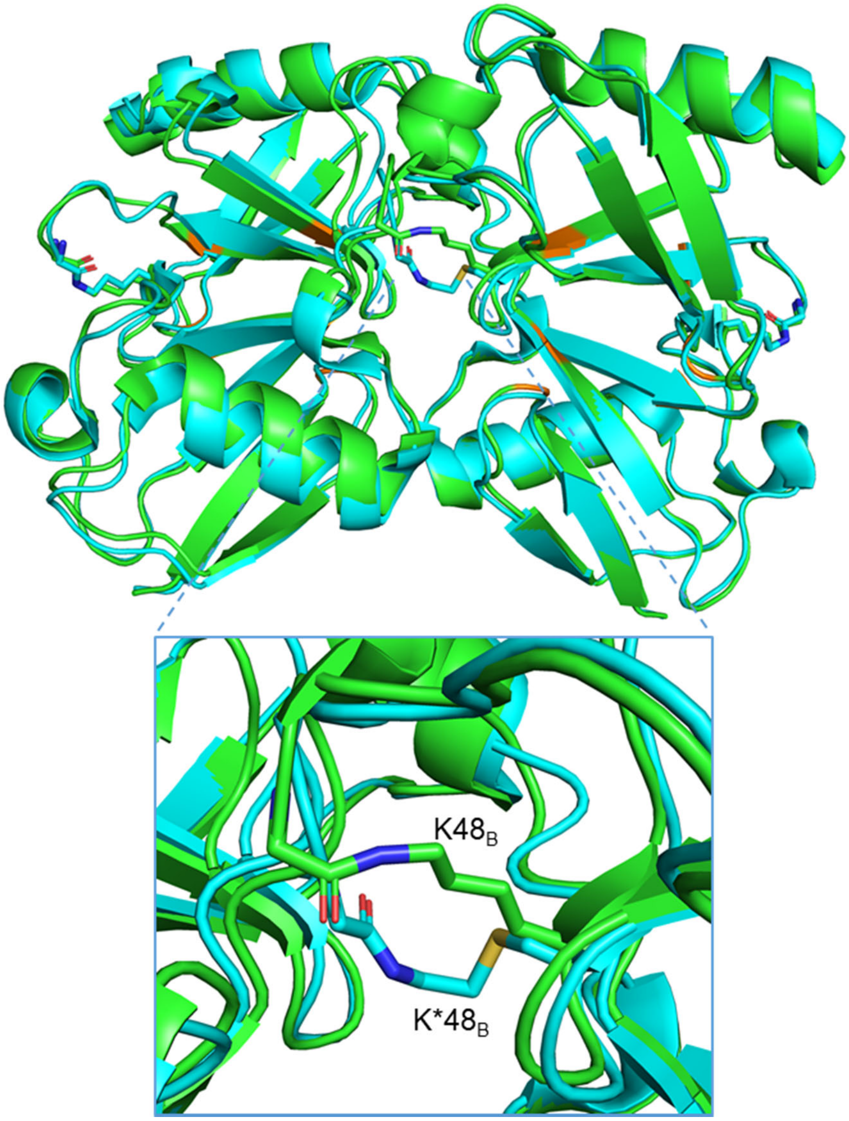

Supplementary Figure 8. Crystal structure of K48-linked tetra-Ub determined in this work (PDB ID 8e7o) and its comparison with the previous structure (PDB ID 2o6v).

(**a**) Cartoon representation of the tetra-Ub structure (PDB ID 8e7o, colored green) with the hydrophobic patch residues of all four Ub units shown as spheres (side chains) colored orange.

(**b**) Stereoview of the fragment of the tetra-Ub structure (PDB ID 8e7o), showing the isopeptide linkage between the C-terminus of the distal Ub (Ub_D) and K48 of the *endo* Ub unit (Ub_C). Shown as blue mesh is the $2mF_o$-DF_c electron density map contoured at 1.5 σ level.

(**c**) Alignment of the tetra-Ub structure determined here (PDB ID 8e7o, colored green) with the previously published structure[1] (PDB ID 2o6v) shown in cyan, the Cα r.m.s.d. is 0.837 Å. The inset below zooms in on the linker region between the two *endo* Ub units (Ub_B and Ub_C) where the largest difference between the two structures is observed. In the tetra-Ub construct of the 2o6v structure lysine-48 was replaced with thialysine (S-Aminoethyl-l-cysteine, marked as K*48), whereas it is a natural lysine (K48) in the tetra-Ub construct used in this work.

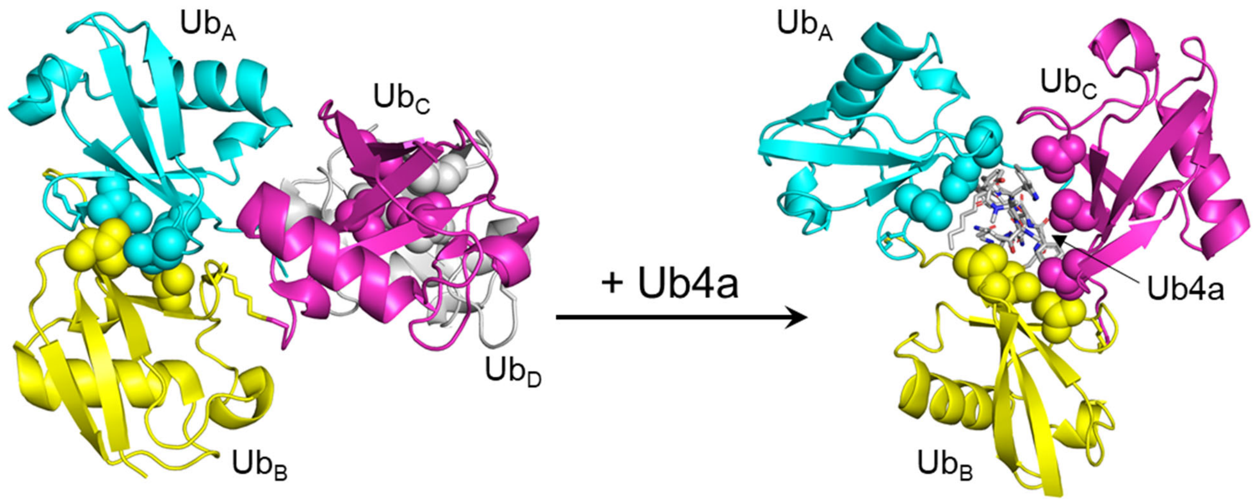

Supplementary Figure 9. Illustration of conformational rearrangements in polyUb upon Ub4a binding. Left: compact conformation of unbound tetra-Ub observed in crystals (from Supplementary Figure 8, panel a, PDB ID 8E7O). Right: Crystal structure of K48-linked tri-Ub in complex with Ub4a (from Figure 5, PDB ID 8F1F). Ub units A-C are colored as in Figure 5; the (distal) Ub$_D$ unit in tetra-Ub is colored light gray. The side chains of hydrophobic patch residues are shown in spheres representation. The Ub4a peptide is shown in sticks representation.

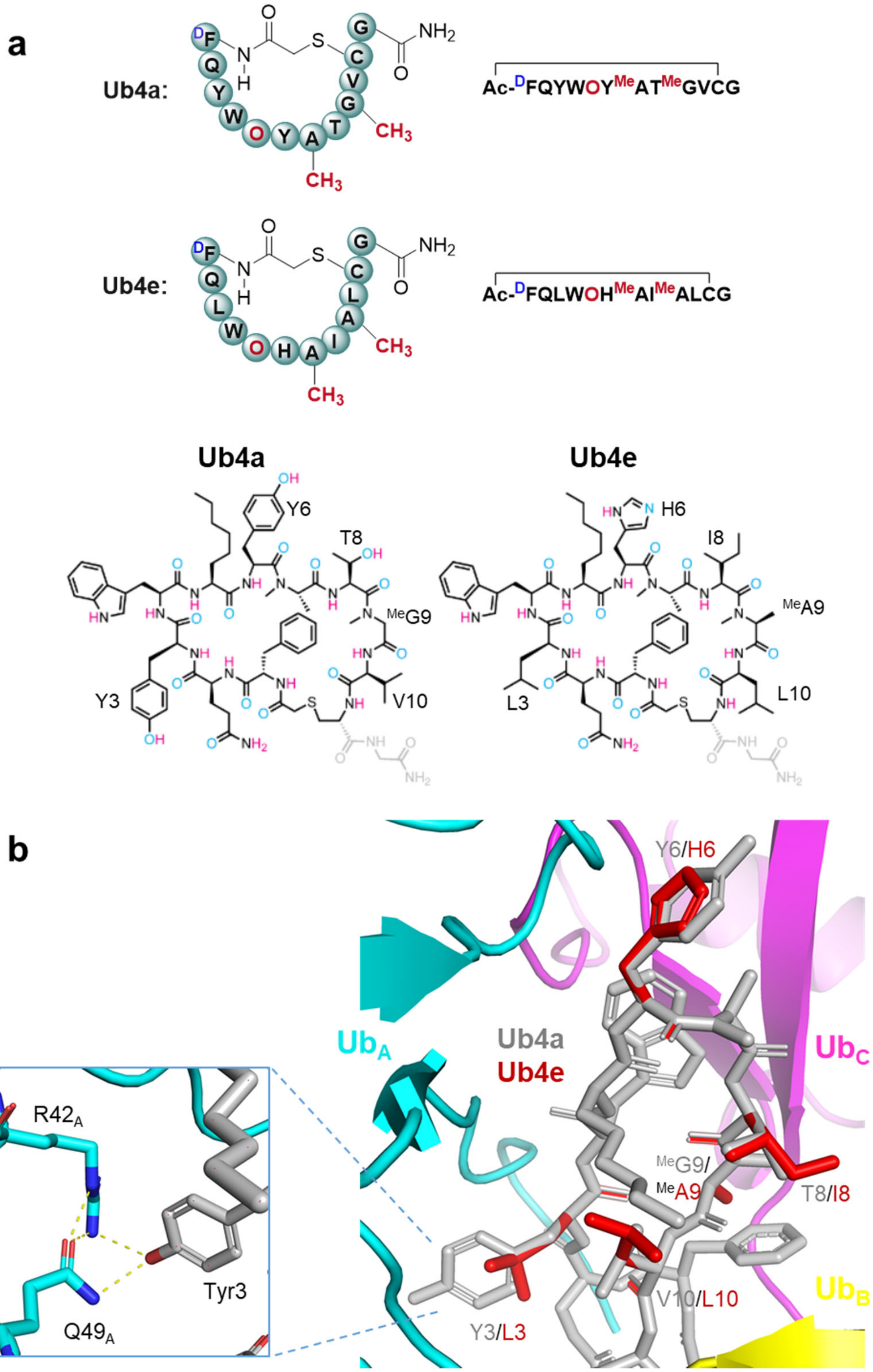

Supplementary Figure 10. Comparison of macrocyclic peptides Ub4a and Ub4e.

(**a**) Comparison of the amino-acid composition and chemical structures of cyclic peptides Ub4a and Ub4e. The five amino acid residues that are different between Ub4a and Ub4e are indicated.

(**b**) Modeled structure of Ub_3:Ub4e complex based on our structure of Ub_3:Ub4a complex, in which the five amino residues, Tyr3, Tyr6, Thr8, ^{Me}Gly9, and Val10, were substituted with Leu3, His6, Ile8, ^{Me}Ala9, and Val10, respectively, using PyMol[2] v2.5.2. The Ub4a peptide is shown in grey, the replacement side chains are in red. Ub units are colored cyan (Ub_A), yellow (Ub_B) and magenta (Ub_C) as in the main text Figures 5-6. The inset shows H-bonds between the OH group of Tyr3 of Ub4a and R42 and Q49 of Ub_A; these interactions are disrupted by the Tyr3 → Leu3 replacement.

Supplementary Table 1. X-ray data collection and refinement statistics for tri-Ub:Ub4a complex and for tetra-Ub.

	**Tri-Ub:Ub4a**	**Tetra-Ub**
**Data collection**		
Space group	P2	$C222_1$
Cell dimensions [&]		
a, *b*, *c* (Å)	83.21, 26.96, 110.47	58.84, 77.16, 135.82
α, β, γ (°)	90.0, 99.9, 90.0	90.0, 90.0, 90.0
Resolution (Å)	30-1.85 (1.88-1.85)*	67.91-1.70 (1.73-1.70)
R_{sym} or R_{merge}	0.062 (0.375)	0.043 (2.000)
$I / \sigma I$	17.3 (2.9)	18.8 (1.7)
Completeness (%)	93.8 (98.9)	99.3 (96.8)
Redundancy	3.1 (3.2)	7.8 (7.3)
**Refinement**		
Resolution (Å)	30.0-1.85	67.91-1.70
No. reflections	37740 / 1986	34167 / 1986
R_{work} / R_{free}	0.228 / 0.298	0.157 / 0.221
No. atoms		
Protein	3631	2406
Ligand/ion	250	20
Water	302	160
B-factors		
Protein	20.5	48.2
Ligand/ion	16.3	186.0
Water	29.3	62.3
R.m.s. deviations		
Bond lengths (Å)	0.020	0.011
Bond angles (°)	2.11	1.19

[&]Number of crystals $n = 1$ for each structure.
*Values in parentheses are for the highest-resolution shell.

Supplementary Table 2. Stabilizing interactions between three Ub units in the tri-Ub:Ub4a complex

Interface	Stabilizing interactions
**Ub_A...Ub_B**	**Covalent bond**: Lys48(NZ)$_A$-Gly76(C)$_B$<br>**H-bonds**: Gly47(O)$_A$-Arg74(N)$_B$ [2.7 Å], Ala46(O)$_A$-Gln40(NE2)$_B$ [3.6 Å]<br>**Hydrophobic contacts**: Ile44(CD1)$_A$-Leu73(CD2)$_B$, Ile44(CD1)$_A$-Leu71(CD2)$_B$
**Ub_B...Ub_C**	**Covalent bond**: Lys48(NZ)$_B$-Gly76(C)$_C$<br>**H-bonds**: Gln49(N)$_B$-Arg74(O)$_C$ [3.0 Å], Gly47(O)$_B$-Arg74(N)$_C$ [3.1 Å]<br>**Hydrophobic contacts**: Ile44(CD1)$_B$-Leu73(CD1)$_C$, Leu8(CD2)$_C$-Ile44(CD2)$_B$
**Ub_C...Ub_A**	**H-bonds**: Arg72(NH1)$_C$-Asp77(OD2)$_A$ [3.0 Å], Arg72(NH1)$_C$-Gly75(O)$_A$ [2.6 Å], Gln49(NE2)$_C$-Arg74(O)$_A$ [3.3 Å], Gln49(O)$_C$-Arg74(NH2)$_A$ [2.8 Å]

Supplementary Table 3. Stabilizing interactions between Ub4a and Ub units of tri-Ub

**Fragment of Ub4a**	Interacting residues from **tri-Ub**
**D-Phe1**	O-atom in two H-bonds with guanidine group of Arg42$_C$; phenyl ring occupying cavity formed by side chains of Leu8$_C$, Val70$_C$, Leu73$_C$, Ile44$_B$ and Val70$_B$
**Gln2**	O-atom forms H-bond with Leu73(N)$_A$, NE2-atom H-bonded to Asp77(O)$_A$
**Tyr3**	OH-function H-bonded with Arg42(NH2)$_A$ and Gln49(NE2)$_A$
**Trp4**	N-atom H-bonded with Leu71(O)$_A$, O-atom H-bonded with Leu71(N)$_A$. Side chain in the cavity formed by Leu8$_A$, Leu71$_A$ and Leu73$_A$, Ile44$_C$, His68$_C$, Val70$_C$
**Aoc5**	Side chain stabilized by His68$_A$, Leu8$_B$ and Leu71$_B$
**Tyr6**	No obvious stabilizing interactions - protrudes from tri-Ub central hole
**N^{Met}-Ala7**	N^{Met}-group interacts with Leu8$_B$
**Thr8**	Side chain wedged between side chains of Leu8$_B$ and Val70$_B$
**N^{Met}-Gly9**	N^{Met}-group interacts with Leu8$_C$
**Val10**	Side chain interacts with side chains of Leu71$_B$ and Val73$_B$
**Linker (TAI)**	S-atom interacts with Leu73$_C$, Val70$_B$ and the hydrophobic section of the side chain from Arg42$_B$. O4-atom forms H-bond with Leu73(N)$_B$.

Supplementary Methods

Synthesis of Ub4a cyclic peptide

The cyclic peptide Ub4a was synthesized as detailed elsewhere[3].

Synthesis of Ub4a cyclic peptide labeled with TEMPO free radical

Fmoc-Solid phase peptide synthesis (Fmoc-SPPS) was carried out on Rink amide resin (0.26 mmol/g, 0.05 mmol scale) for the synthesis of the Ub4a cyclic peptide labeled with TEMPO free radical. All amino acids were coupled at room temperature by using amino acid (4.0 equiv), DIEA (8.0 equiv) and HCTU (4.0 equiv). Cys (Acm) was incorporated at C-terminus of the sequence. Automated peptide synthesizer was used to couple all the amino acids in the sequence. Fmoc deprotection was performed with 20% piperidine. The N-Methylation at the specific sites in the peptide sequence was performed manually.

The following steps were involved in the N-Methylation.

1. The peptide-resin with free N-terminus was sequentially washed with N, N-dimethylformide (DMF) and N-methylpyrrolidone (NMP). Next, the resin was treated with a solution of o-nitrobenzene sulfonyl chloride (o-NBS) (44.5 mg, 4.0 equiv) in 1 mL of NMP and sym-collidine (66.0 µl, 10.0 equiv) at room temperature and was shaken for 20 minutes. Then the resin was washed with NMP (3 X 2.5 mL) twice.

2. The resin was washed with dry tetrahydrofuran (THF) (3 X 2.5 mL). Then, the resin was treated with a solution of triphenylphsophine (PPh3) (65.6 mg, 5.0 equiv) in 1.43 mL of dry THF, anhydrous methanol (~20 µl, 10.0 equiv) at room temperature and shaked it for 2 minutes. To the 0.5 mL of dry THF diisopropyl azodicarboxylate (DIAD) (~49.3 µl, 5.0 equiv) was added and divided into 5 portions. After 10 minutes interval, each portion was treated with resin and was shaken at room temperature. Next, wash the resin with THF (3 X 2.5 mL) twice.

3. The resin was washed with NMP (3 X 2.5 mL). Then the resin was treated with a solution of 2-mercaptoethanol (35 µl, 10 equiv) in 1 mL of NMP, 1, 8-Diazabicyclo[5.4.0] undec-7-ene (DBU) (37.8 µl, 5.0 equiv) at room temperature and was shaken for 15 minutes. The resin was washed with NMP (3 X 2.5 mL) twice. Next, the resin was washed with DMF (3 X 2.5 mL).

The next amino acid after N-Methylation was coupled using HATU (4.0 equiv), DIEA (8.0 equiv) for 1 h (2 X). Chloroacetic acid (70.9 mg, 15.0 equiv) was coupled at N-terminus using N, N'-diisopropylcarbodiimide (DIC) (~78.3 µl, 10 equiv) in 1 mL of DMF. Then global deprotection was performed with a mixture of TFA/ TIPS/ H2O (95.0:2.5:2.5) followed by precipitation and lyophilization. Then cyclization step was performed in 6M Gn.HCl at pH 8.0 (adjusted with NaOH) and incubated at 37 ^{o}C. Analytical HPLC was performed using a C18 column with a gradient of 0-60% B over 30 min. After completion of cyclization, 4h, the crude peptide was purified using a C18 preparative column with a gradient flow of 0-60% B over 60 min give peptide 2 in 48% yield (see Supplementary Figure S5).

Acm deprotection [4]. $PdCl_2$ (5.1 mg, 10.0 equiv) was dissolved in 6M Gn·HCl/200 mM phosphate buffer (pH 7.3, 50 µL) at 37 °C for 20 min. 6M Gn.HCl/200 mM phosphate buffer (pH 7.3, 2858.5 µL, 2 mM) was added to the peptide 2 (5.0 mg, 5.72 mmol, 1.0 equiv) at room temperature. The prepared PdCl2 solution was added to the peptide solution, and the mixture

was incubated at 37 °C. An aliquot of the crude reaction mixture was quenched with DTT and injected into the HPLC using a C18 analytical column with a gradient of 0-60% B in 30 min. After completion of the reaction, 1 h, the reaction crude was purified using a C18 semi-preparative column with the gradient flow of 0-60% B over 60 min give peptide 3 in 42% isolated yield (2.0 mg).

Labeling with TEMPO, free radical. The peptide 3 (2.0 mg, 1.19 mmol, 1.0 equiv.) in 6M Gn.HCl/200 mM phosphate buffer (pH 7.3, 1193 µL, 1 mM) was cooled to 0 °C for 5 min. A solution of TEMPO (4-maleimido-2,2,6,6-tetramethyl-1-piperidinyl oxy) free radical (1.35 mg, 4.5 equiv.) in 50 µl of DMF at 0 °C was prepared under dark and this was added to peptide solution under dark conditions, and the reaction was kept at the same temperature. Analytical HPLC was performed using a C4 column with a gradient of 0-60% B over 30 min. The reaction was completed in 1h. The crude peptide was purified using a C4 semi-preparative column with a gradient flow of 0-60% B over 60 min gave peptide 4 in 68% isolated yield (1.56 mg) (see Supplementary Figure S5).

Supplementary references

1 Eddins, M. J., Varadan, R., Fushman, D., Pickart, C. M. & Wolberger, C. Crystal Structure and Solution NMR Studies of Lys48-linked Tetraubiquitin at Neutral pH. *J Mol Biol* **367**, 204-211 (2007).

2 *The PyMOL Molecular Graphics System, Version 2.5, Schrodinger, LLC* (2015).

3 Rogers, J. M. *et al.* In vivo modulation of ubiquitin chains by N-methylated non-proteinogenic cyclic peptides. *RSC Chemical Biology* **2**, 513-522 (2021).

4 Maity, S. K., Jbara, M., Laps, S. & Brik, A. Efficient Palladium-Assisted One-Pot Deprotection of (Acetamidomethyl)Cysteine Following Native Chemical Ligation and/or Desulfurization To Expedite Chemical Protein Synthesis. *Angew Chem Int Ed Engl* **55**, 8108-8112 (2016).
